# No Difference between the Efficacy of High-Nitrate and Low-Nitrate Vegetable Supplementation on Blood Pressure after 16 Weeks in Individuals with Early-Stage Hypertension: An Exploratory, Double-Blinded, Randomized, Controlled Trial

**DOI:** 10.3390/nu16173018

**Published:** 2024-09-06

**Authors:** Dandan Li, Elena Jovanovski, Andreea Zurbau, John Sievenpiper, Davor Milicic, Ahmed El-Sohemy, Vladimir Vuksan

**Affiliations:** 1 Department of Nutritional Sciences, University of Toronto, 1 King’s College Circle, Toronto, ON M5S 1A8, Canada; dandan.li@mail.utoronto.ca (D.L.);; 2 Clinical Nutrition and Risk Factor Modification Centre, St. Michael’s Hospital, Unity Health Toronto, 30 Bond Street, Toronto, ON M5B 1X1, Canada; 3 Department of Cardiovascular Diseases, School of Medicine, University of Zagreb, University Hospital Center Zagreb, Kispaticeva 12, 10000 Zagreb, Croatia

**Keywords:** dietary nitrate, nitric oxide, vegetable supplement, blood pressure, arterial stiffness, cardiovascular disease risk factors, heart-healthy diet

## Abstract

Dietary inorganic nitrate lowers blood pressure (BP) in healthy individuals through improved nitric oxide (NO) bioavailability. However, there is limited evidence examining the long-term effects of dietary nitrate for managing hypertension. We aimed to determine whether the sustained intake of dietary nitrate improved BP and cardiovascular disease (CVD) risk factors in individuals with early-stage hypertension. The Dietary Nitrate (NO_3_) on BP and CVD Risk Factors (DINO_3_) Trial was a multi-center, double-blinded, parallel, randomized, controlled trial in participants with elevated BP. Participants were supplemented with high-nitrate (HN) (~400 mg nitrate) or low-nitrate (LN) vegetable powder (~50 mg nitrate) on top of their usual diets for 16 weeks. The primary outcome was office systolic BP at 16 weeks. The secondary outcomes were 24 h ambulatory BP, central BP, heart-rate-corrected augmentation index (AIx75), carotid–femoral pulse wave velocity (cf-PWV), lipids, and high-sensitivity C-reactive protein (hs-CRP). Sixty-six participants were randomized at baseline (39M:27F, age: 51.5 ± 10.8 years, BMI:27.9 ± 3.2 kg/m^2^). In an intention-to-treat analysis, no differences were observed between HN and LN groups in terms of office systolic BP at 16 weeks (3.91 ± 3.52 mmHg, *p* = 0.27) or secondary outcomes. In this exploratory study, sustained HN vegetable supplementation did not exhibit more favorable vascular effects than LN vegetable supplementation in individuals with elevated BP.

## 1. Introduction

Dietary nitrate has garnered significant interest for its cardioprotective benefits. Nitrate in our food supply is present in the inorganic form (NO_3_^−^) and is concentrated in beetroot and green leafy vegetables such as spinach, arugula and kale. It has been suggested that dietary nitrate plays an important role in the blood pressure (BP)-lowering effects of the Dietary Approach to Stop Hypertension (DASH) diet [1], which incorporates 8 to 10 servings of vegetables per day. Moreover, dietary nitrate could represent an important bioactive linking the benefits observed with the consumption of green leafy vegetables and cardiovascular disease (CVD), which show strong inverse associations over long-term intake [2]. Despite advancements in antihypertensive pharmacological treatments and the demonstrated success of numerous dietary patterns, high BP remains a highly uncontrolled condition and represents the leading risk factor driving mortality rates globally [3]. Targeted and achievable dietary strategies to manage BP are needed.

Webb et al. showed that the ingestion of a single dose of beetroot juice providing 1395 mg nitrate in healthy subjects reduced systolic BP by 10 mmHg compared to water, with peak reductions occurring at 2.5 h [4]. These effects coincided with an increase in plasma cyclic guanosine monophosphate (cGMP) levels, which suggested the activation of soluble guanylate cyclase (sGC) via nitric oxide (NO). Govoni et al. [5] and Webb et al. [4] further demonstrated that the administration of an antibacterial mouthwash or expectorating saliva abolished the rise in plasma nitrite and vascular response. It is now generally understood that the enterosalivary circulation plays an important role in the reduction of nitrate to nitrite in the oral cavity and in the eventual systematic conversion of nitrite to NO. The characterization of this pathway, which generates NO independently from the enzymatic activity of nitric oxide synthase (NOS) and its substrate L-arginine, has illuminated the vast potential of dietary nitrate to augment NO bioavailability.

Despite encouraging findings from the assessment of acute responses, the chronic supplementation of dietary nitrate on nitrate–nitrite metabolism and BP is less well understood. Results from longer-term studies have also been mixed, particularly for those with elevated CVD risk [6,7]. In one of the few studies that tested the effect of beetroot juice over 12 weeks, the percent conversion of nitrate to nitrite remained consistent throughout the study in healthy individuals, indicating a lack of tolerance from dietary nitrate—a major limitation of pharmacological organic nitrates [8]. In a hypertensive patient group, however, nitrate-rich green leafy vegetables did not significantly raise plasma nitrite levels after 5 weeks, despite raising plasma nitrate three-fold. This coincided with a lack of improvement in clinic and ambulatory BP compared to the low-nitrate control [9], conflicting with an earlier study showing substantial BP reductions from beetroot juice only [10]. In this study, robust reductions in clinic BP were observed alongside benefits to home and ambulatory measures in hypertensive patients. This was further supported by significant improvements in endothelial function and arterial stiffness, indicating promising functional and structural changes in the cardiovascular system after 4 weeks of supplementation [10]. While dietary nitrate appears to benefit several related but independent risk factors of CVD, clinical trials beyond 4 weeks are scarce, and other vegetable sources besides beetroot have not been extensively studied.

Therefore, our primary objective was to determine whether high-nitrate (HN) vegetable supplementation compared to low-nitrate (LN) vegetable supplementation lowers office systolic BP after 16 weeks in those with early-stage hypertension. Additional CVD risk factors were also assessed, including ambulatory BP, arterial stiffness, serum lipids, and high-sensitivity C-reactive protein (hs-CRP).

## 2. Materials and Methods

### 2.1. Ethics

The trial was registered on clinicaltrials.gov (NCT03478631) and reported according to CONSORT (Consolidated Standards of Reporting Trials). The study was approved by the research ethics boards of St. Michael’s Hospital and University of Zagreb School of Medicine and was carried out in accordance with Good Clinical Practice guidelines.

### 2.2. Study Design

The Dietary Nitrate on Blood Pressure (DINO3) study was a randomized, double-blinded, parallel study with two experimental arms of 16-week duration. Participants were randomly assigned to supplement their usual diet with HN or LN vegetable powder under free-living conditions. Randomization lists were created by a laboratory member who was otherwise not involved in the study, using a computer-generated random number generator, and participants were stratified by sex and baseline age to ensure balanced comparisons. Random permuted blocks of varying sizes (≤10) were used to eliminate the possibility of predicting a future allocation leading to selection bias. Treatment allocation was revealed to the study coordinator after stratum entry, in the presence of the participant during the baseline visit, on a patient-by-patient basis. Vegetable powders were provided in coded, identical opaque bottles, which allowed for the blinding of treatments to participants and study personnel. Participants were provided with the vegetable powders at baseline and attended follow-up visits for clinic measurements at 8 and 16 weeks.

### 2.3. Study Participants

In this multi-center study, participants were recruited from urban areas in Toronto, Canada, and Zagreb, Croatia. In Zagreb, participants were recruited primarily via family physicians at a local hospital and an existing patient database. In Toronto, participants were recruited from primary care physicians at St. Michael’s Hospital, internet advertisements, local newspapers, subway advertisements, and an existing database of individuals who previously expressed interest in participation. Individuals were initially screened on the telephone and then invited to the clinic for a detailed information session. Those who continued to show interest provided written consent and underwent a screening procedure, which involved anthropometric measurements (height, body weight, body fat%), a detailed report of their medical history, office BP measurements and a fasted venous blood draw for serum triglycerides, AST and creatinine. Enrolled participants were men and women between 30 and 65 years of age with a resting clinic systolic BP between 130 and 160 mmHg and a BMI between 18.5 and 35kg/m^2^. Individuals were excluded if they were taking antihypertensive medication or supplements/herbs affecting blood pressure or were prescribed nonsteroidal anti-inflammatory drugs, antacids, warfarin, and medications affecting NO synthesis (sildenafil, organic nitrates, etc.). Participants must not have taken antibiotics within the past 3 months, had a major cardiovascular event in the past year (stroke or myocardial infarction), experienced chest pain, been diagnosed with a medical condition requiring continuous medical attention, or had a serum triglyceride level > 4.5 mmol/L. Participants were excluded if they reported consuming greater than three servings of vegetables per day based on a semi-quantitative food frequency questionnaire, smoked, or had more than two drinks of alcohol per day. Participants had to be willing to refrain from using mouthwash and consuming non-plant foods rich in nitrate or nitrite throughout the study. Furthermore, participants were asked to maintain a constant level of physical activity and adhere to their usual lifestyle throughout the study.

### 2.4. Dietary Interventions

Participants were provided with a 16-week supply of HN or LN dehydrated vegetable powder to incorporate into their regular diet. A daily dose of ~400 mg nitrate in the HN group was chosen based on its previously demonstrated efficacy in individuals with elevated BP [10] and on its feasibility as an amount that could be consumed through a reasonable number of vegetable servings. Participants were asked to take 30 g (about 4.5 tablespoons) of powder daily, which provided the equivalent of seven or eight servings of vegetables. Participants were instructed to consume 10 g of powder with a meal, three times a day, either incorporated into water or food. The HN vegetable blend comprised 60% beetroot, 28% kale and 12% spinach. The LN vegetable blend comprised 35% tomato, 25% broccoli, 28% carrot and 12% peas and provided less than 50 mg of nitrate per day. Vegetable powders were formulated specifically for this trial and were prepared courtesy of FutureCeuticals (Momence, IL, USA). The drying of the individual vegetables was achieved either through freeze-, air- or spray-drying, which retained the nutrient profile of the vegetables. Table 1 provides the nutritional composition of a daily serving of HN and LN supplements based on laboratory chemical analysis.

### 2.5. Outcome Measurements

Participants were asked to attend all study visits after fasting for 10–12 h prior to their study appointment time, which was between 07:00 and 11:00 AM.

#### 2.5.1. Office Blood Pressure

Office brachial BP was measured at each visit using an automated BP monitor (OMRON Intellisense HEM-907, Omron Healthcare, Kyoto, Japan) in triplicate in 1 min intervals. The participant was seated in a quiet and dark room, with the measurement taken after 5 min of rest. The BP cuff was placed on the participant’s non-dominant arm. The displayed average of three readings was used and measured accurately according to ACC/AHA guidelines [11]

#### 2.5.2. Ambulatory Blood Pressure

Ambulatory 24 h BP was measured with a portable Spacelabs 90207 device (Spacelabs, Bellevue, WA, USA) at baseline and 16 weeks. Automatic pressure readings were taken over 24 h at 30 min intervals during wake periods and every 60 min during sleep periods. Wake and sleep hours were adjusted according to the participant’s sleep schedule. Cuff sizes were determined based on the participant’s arm circumference, and cuffs were fitted on the non-dominant arm.

#### 2.5.3. Central Blood Pressure and Arterial Stiffness

Changes in arterial physiology were assessed through measures of central BP and the central augmentation index at all study visits and carotid–femoral pulse wave velocity (cf-PWV) at the baseline and end visits. Cf-PWV was measured non-invasively via applanation tonometry using the Sphygmocor Vx system (AtCorMedical Inc., Sydney, Australia). The velocity of the BP pulse waveform along an arterial segment was calculated by capturing the pulse pressure waveform at two superficial artery sites, specifically the carotid and femoral artery. An ECG signal was simultaneously recorded with the pulse pressure to provide R-wave timing reference. The Sphygmocor system utilized the collected data to calculate the mean time difference (Δt) between the R-wave and the pressure wave per heartbeat. The Δt and the measured length of the arterial segment was utilized to calculate PWV. Estimates of central BP and AIx were derived from radial artery waveforms utilizing a validated transfer function [12,13], and they were based on an average of three high-quality recordings. Given the inverse and linear relationship between heart rate and AIx [14,15,16], AIx was normalized to a heart rate of 75 beats per minute according to the following equation: AIx75 = −0.48 × (75 − HR) + AIx [17].

#### 2.5.4. Additional CVD Risk Factors

At the baseline and end visits, serum hs-CRP and serum lipids (Total-C, LDL-C, HDL-C) were measured, given their association with CVD risk [18,19]. Fasting blood samples were drawn passively from the median-cubital vein by trained phlebotomists. Serum hs-CRP and cholesterol levels were analyzed by the core laboratory facility at St. Michael’s Hospital in Toronto using routine analytical methodologies.

#### 2.5.5. Compliance and Safety

Height was measured with a wall-mounted stadiometer (Perspective Enterprises, Portage, MI, USA). At all visits, body weight was assessed by a beam scale, body fat composition was measured by bioelectrical impedance (Body Composition Analyzer “Tanita”, Tanita Corp. of America Inc., Arlington Heights, IL, USA), and BMI was calculated with body weight and height (kg/m^2^). The remaining powder was weighed in the bottle to determine adherence as a percentage of total provided powder while maintaining blinding. At the final visit, nutrient intake from foods were estimated from three-day food records completed for the three days prior to the study visit. A 24 h urinary collection was also completed over 24 h prior to the final study visit to determine 24 h sodium and potassium excretion (mmol/d). Serum AST and creatinine were assessed at every study visit to monitor signs of liver and kidney damage. Any occurrence of adverse reactions or events was monitored throughout the trial through the review of symptom diaries at every study visit and frequent communication between participants and study coordinators.

#### 2.5.6. Statistical Analyses

Baseline characteristics were compared between treatment groups using the chi-squared test and Student’s *t*-test for categorical and continuous variables, respectively. For the primary analysis, ANCOVA was performed to determine the effect of treatment on outcomes at 16 weeks, with adjustment for center, baseline value, age, sex, and BMI. Multiple imputation was used to fill in missing data in the intention-to-treat (ITT) analysis. In secondary analyses, repeated-measures analysis was performed using all available data from participants randomized at baseline [20], to test the effect of treatment on vascular outcomes over 0, 8 and 16 weeks. A linear mixed model was utilized to determine the fixed effects of treatment and treatment–time interaction on outcome measures, with adjustment for age, sex, center and BMI. A covariance structure was modeled for all measurements from the same participant to represent random effects. Tests for treatment effects at 16 weeks and repeated-measures analyses were also performed for completers only. Pre-specified subgroup analyses (age, sex, center, BMI and baseline value) were performed for completers with ANCOVA after adjustment for center and baseline values. A term denoting the subgroup of interest was included along with a treatment x subgroup interaction term with its corresponding *p*-value < 0.05, to determine the significance of the subgroup effect. Analyses were conducted using SAS OnDemand for Academics (previously SAS University Edition). Data are presented as means ± SE unless indicated otherwise.

## 3. Results

### 3.1. Baseline Clinical Characteristics

**Figure 1:**
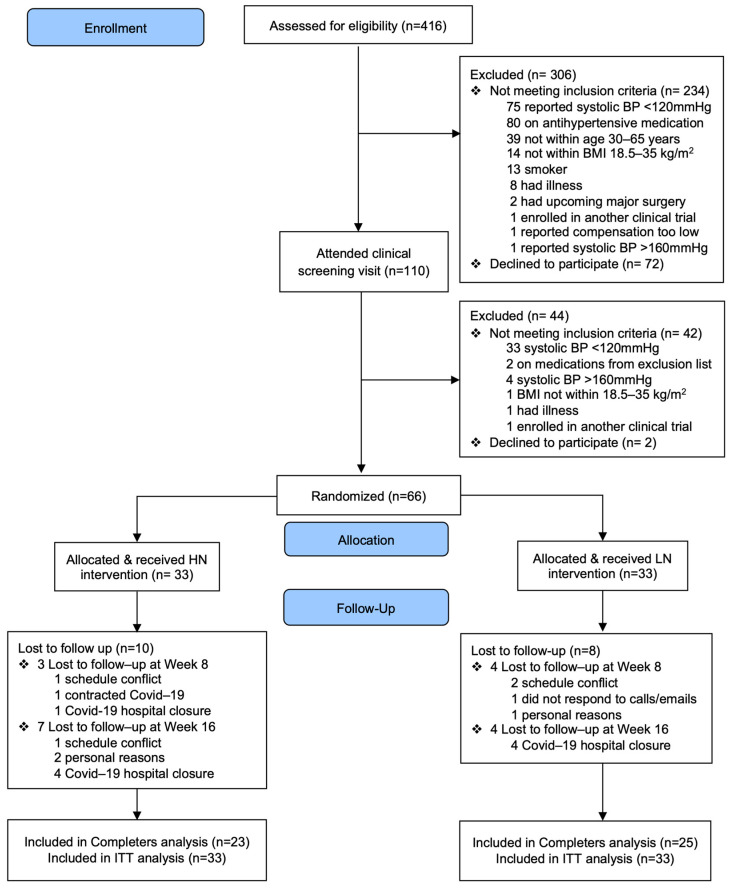
Flow of participants in the study.

Participant recruitment and data collection occurred from March 2019 to February 2020, and March 2021 to November 2021. A total of sixty-six participants were randomized to the HN or LN treatment. The attrition rate and reasons for missing data were similar between the two groups. The flow of participants throughout the study is presented in Figure 1.

Baseline characteristics of randomized participants are shown in Table 2. On average, participants were middle-aged (mean age: 51.5 ± 10.8 years) and overweight (mean BMI = 27.9 ± 3.2 kg/m^2^, mean body fat in females = 36.7%, mean body fat in males = 24.7%), and 59% of participants were male and 41% were female. Participants had normal cholesterol levels [21] and above-normal hs-CRP levels (mean hs-CRP = 2.03 ± 2.4 mg/L) [22]. Participants had stage I/stage II hypertension (mean office BP = 138.6 ± 10.5 mmHg systolic and 86.9 ± 8.8 mmHg diastolic; mean ambulatory BP = 130.5 ± 10.4 mmHg systolic and 81.8 ± 7.9 mmHg diastolic) and did not take antihypertensive medication. Participants had been diagnosed or aware that they had elevated blood pressure for roughly 4 years (47.9 ± 99.6 months). The range of elevated BP duration was 0–480 months, which included individuals who were not aware they had elevated BP until the screening visit and people who had had elevated BP for most of their life. Indices of arterial stiffness represent values observed in low-CVD-risk populations (mean AIx75 = 23.7 ± 10.7; mean cf-PWV = 7.5 ± 3.0) [23,24]. There were no significant differences in clinical characteristics between groups at baseline, except for serum creatinine levels (*p* = 0.02).

### 3.2. Treatment Effects on Blood Pressure

**Figure 2:**
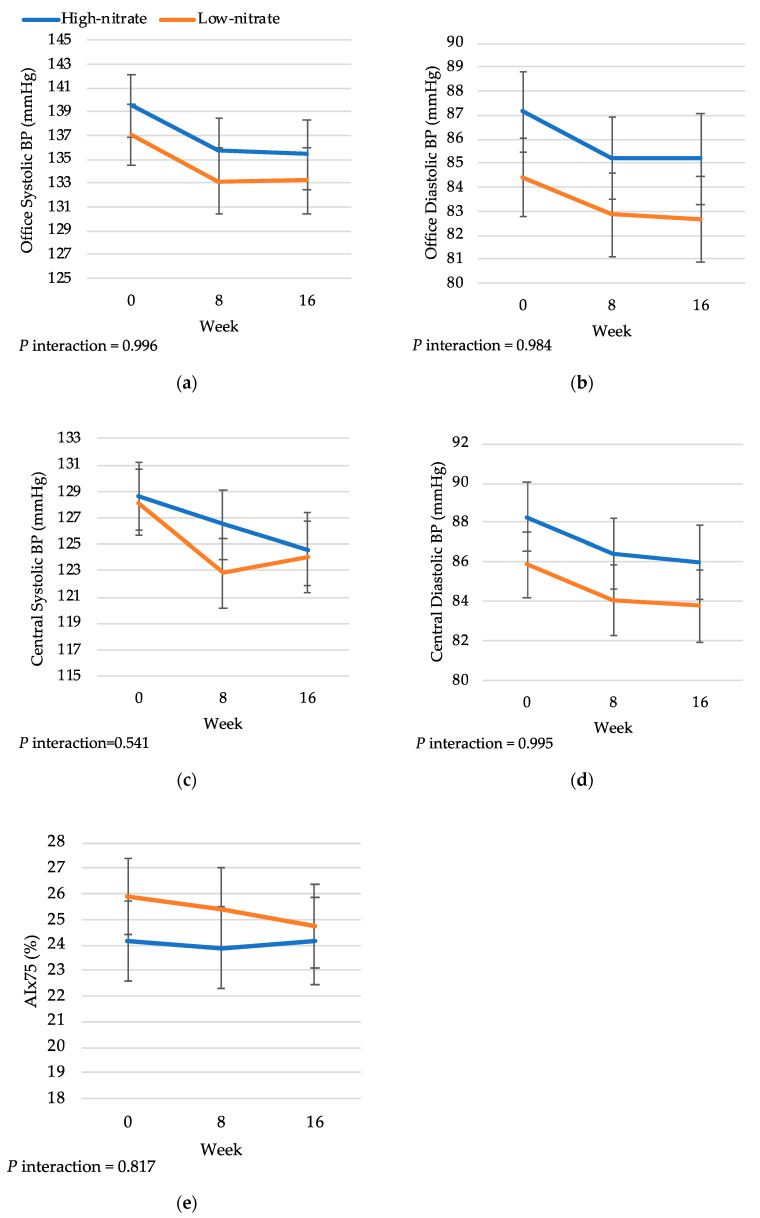
Changes in (**a**) office systolic BP, (**b**) office diastolic BP, (**c**) central systolic BP, (**d**) central diastolic BP, and (**e**) AIx75 in HN and LN group from baseline to 8 weeks and 16 weeks. *p*-values for test of treatment-by-time interaction from a linear mixed model adjusted for age, sex, center, BMI. Analysis includes all available data from participants randomized at baseline (n = 66), with n = 66 for all outcomes at week 0. Data were available for n = 56 and n = 48 for week 8 and 16, respectively, for office systolic BP and office diastolic BP; n = 52 and n = 47 for week 8 and 16, respectively, for central systolic BP and central diastolic BP; and n = 54 and n = 47 for week 8 and 16, respectively, for AIx75. Data represented as means ± SE.

**Figure 3:**
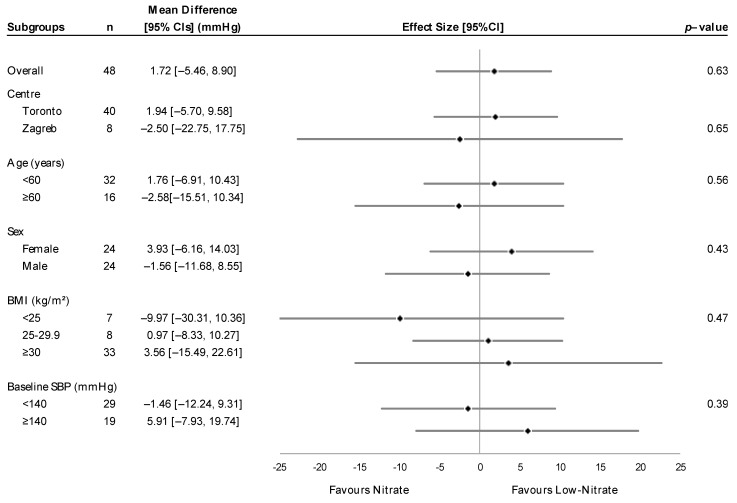
Subgroup analysis for the effect of HN compared to LN treatment on office SBP, in completers (n = 48). Mean differences with 95% CIs represent the treatment difference between groups within each subgroup category, adjusted for center and baseline values, unless not applicable. *p*-values represent test for treatment x subgroup interaction from ANCOVA. BMI, body mass index; CI, confidence interval; SBP, systolic blood pressure.

In the ITT analysis, there was no overall significant difference between HN and LN treatments on office systolic or diastolic BP at 16 weeks (3.91 ± 3.52 mmHg, *p* = 0.27 and 2.21 ± 2.09 mmHg, *p* = 0.30, respectively) (Table 3). Individually, the LN group showed a near-significant reduction in office systolic BP (−4.39 ± 2.19, *p* = 0.05), whereas the HN group alone showed no reduction in office systolic BP (−1.30 ± 2.92 mmHg, *p* = 0.66). Similarly, there was no significant difference between HN and LN treatment on central BP, although there was a near-significant reduction in central systolic (−4.17 ± 2.33, *p* = 0.08) and diastolic BP (−2.48 ± 1.31, *p* = 0.06) in the LN group. Similar to office BP, there was no reduction in central systolic and diastolic BP in the HN group alone (−3.84 ± 2.64, *p* = 0.15 and −2.76 ± 2.34, *p* = 0.25, respectively). In ambulatory BP measures, no treatment differences were observed for 24-h, wake or sleep systolic and diastolic BP. Within-group changes further showed non-significant effects (*p* > 0.05). Assessing office and central BP across three study visits, there was no interaction effect between treatment and time, using all available data from participants randomized at baseline (Figure 2). Analyses in completers similarly did not show any significant treatment effects at 16 weeks, or significant treatment-by-time interaction effects on BP measures. Treatment differences in systolic BP according to a priori subgroups in completers are presented in Figure 3. There was no indication of any effect modification according to center, age, sex, BMI and baseline systolic BP (*p* for interaction > 0.05). No subgroup effects were observed for office diastolic BP, ambulatory BP, and central BP values (Figures S1–S5).

### 3.3. Treatment Effects on Arterial Stiffness

HN treatment did not significantly improve cf-PWV (−0.061 ± 0.73 m/s, *p* = 0.93) or AIx75 (−0.26 ± 1.62%, *p* = 0.87) compared to LN treatment in ITT analysis (Table 3). In the repeated-measures analysis of AIx75 over the three study timepoints, there was no significant interaction effect of treatment and time (Figure 2). Completers’ analysis similarly showed no significant treatment effects or treatment-by-time interaction effects. In the analysis of subgroup effects on cf-PWV and AIx75, there was no evidence of effect modification (Figures S6 and S7).

### 3.4. Treatment Effects on Lipids and C-Reactive Protein

No treatment differences were observed for serum total cholesterol, LDL-cholesterol, HDL-cholesterol, non-HDL cholesterol, triglyceride levels, and hs-CRP (*p* > 0.05) in the ITT analysis (Table 3). No significant within-group changes were observed. Completers’ analysis similarly showed no significant treatment effects.

### 3.5. Adherence to Treatments

Adherence to the HN and LN treatments based on the weight of returned supplement containers was 87.49% ± 3.73 and 92.52% ± 3.58, respectively (*p* = 0.33) (Table 4). Vegetable powders were generally well-tolerated. Participants reported the incorporation of the vegetable powder into water, soft drinks, and recipes such as scrambled eggs, salad dressing and baked goods. Body weight did not significantly change in the HN group (0.60 ± 0.44, *p* = 0.18) or in the LN group (−0.54 ± 0.63, *p* = 0.40), and there was no difference between groups in terms of body weight at the end of the study (*p* = 0.14). Throughout the study, four participants initiated antihypertensive medication in the HN group, while one participant initiated antihypertensive medication in the LN group. A chi-square test for independence did not indicate a relationship between the treatment group and the initiation of antihypertensive medication (*p* = 0.16). Moreover, 24 h urinary sodium and potassium excretion (mmol/d), an estimate of 24 h sodium intake, was not significantly different between groups at 16 weeks. Converted to mg/day, the mean sodium intakes were 3937 ± 505 mg and 2934 ± 340 mg per day in the HN and LN groups, respectively. The estimated potassium intakes were 2686 ± 301 mg and 2830 ± 386 mg per day in the HN and LN groups. Estimates of daily caloric and nutrient intake from three-day food records collected at 16 weeks are provided in Table S1. No significant differences between HN and LN groups were detected in macronutrient intake as a percentage of daily total caloric intake, or in the daily intake of minerals and micronutrients.

### 3.6. Adverse Effects

No serious adverse events were reported. Throughout the study, individual serum AST and creatinine remained in normal ranges (7–40 IU/L AST; 50–110 µmol/L creatinine). There were no reports of hypotension. Mild gastrointestinal discomfort was reported, including bloating, which was reported in four participants (LN group), and flatulence, which was reported in seven participants (six in the LN group and one in the HN group). Transient diarrhea was reported in four participants when vegetable powder was consumed on an empty stomach at the start of the intervention or taken without food (one in the LN group and three in the HN group). Transient headache was reported in two participants (one in the LN group and one in the HN group).

## 4. Discussion

In this multi-center, double-blinded RCT, we examined the effect of HN versus LN supplementation on BP and CVD risk factors in sixty-six participants with early-stage hypertension. No difference in the primary outcome (office systolic BP) was observed between groups ingesting vegetable supplements providing either ~400 mg or ~50 mg nitrate after 16 weeks. Additionally, there were no differences in ambulatory BP measures or markers of arterial stiffness as assessed in the pulse wave analysis. Vegetable powders represented a convenient medium to reach the intended dietary nitrate dose and were generally well-tolerated in both groups. Our findings did not support the hypothesis that adding nitrate-rich vegetables to habitual diets for an extended period of 16 weeks would improve BP and arterial stiffness compared to consuming low-nitrate vegetables in individuals with early-stage hypertension.

Our findings showing no difference between a HN and LN vegetable intake on BP measures are consistent with recent publications in individuals with elevated CVD risk [25,26,27,28,29]. Several short-term studies (≤2 weeks) that administered a similar dose of 400mg of nitrate showed no benefit of dietary nitrate to resting BP [27,29] and ambulatory BP [28] in participants with type 2 diabetes or hypercholesterolemia. Despite observations of elevated plasma nitrite levels, which indicate greater NO bioavailability, biochemical perturbations characteristic of type 2 diabetes and inflammatory conditions may attenuate the vascular benefits through greater NO scavenging in an environment of increased oxidative stress [30]. Although participants in our trial were enrolled based on hypertension, a proportion of participants had existing comorbidities including type 2 diabetes (n = 7) and hypercholesterolemia (n = 8). A higher dose of 700mg nitrate daily similarly did not improve clinic BP after a 6-month trial in patients with or at risk of type 2 diabetes, despite decreasing left ventricular volumes indicating benefits to cardiac structure that are independent from BP [31]. Improvements to central hemodynamics were not observed in our study, although further research may be needed since it was not the primary outcome in our study.

Our results support previous pilot trials showing no effect of inorganic nitrate on office/clinic systolic BP in overweight or obese individuals [32,33,34], given that most participants in our study were overweight or obese. In our pre-specified subgroup analysis, we did not observe any trends between systolic BP response and BMI (*p* = 0.47), although a larger sample size would be needed to improve the precision of these estimates. Large variabilities in clinic BP responses were also observed in the trial by Lara et al., which administered a higher dose of ~600 mg/day to thirty participants and found no significant differences in clinic BP [34]. Similarly, they did not find any associations between systolic BP changes and BMI (*p* = 0.09); rather, they found a strong association with age (*p* = 0.006). In our study, participants were between 30 and 65 years of age, with 65% of participants ≥50 years of age. Efficacy findings in older age groups are mixed, with several studies showing no effect [26,35] and others showing elevations in plasma nitrate/nitrite and corresponding BP reductions comparable to younger age groups. Aging is associated with increased arterial stiffening and attenuated vascular reactivity [36], which may influence responsivity to NO. The greater production of reactive oxygen species (ROS) in aging endothelial cells [37] may inactivate NO and therefore play a role in the observed lack of vascular response. Differences in oral microflora in older adults [38] could also interfere with the non-enzymatic multi-step pathway that is critical for NO generation from the ingestion of dietary nitrate. Our study was limited by the lack of plasma and salivary nitrate/nitrite measurements to explore these trends.

The influence of dietary sodium on BP at the final visit should not be overlooked, given that the study design did not restrict sodium intake. The mean 24 h urinary sodium excretion indicated a high sodium intake (3.9 g/d) at the final visit in the HN group and an intermediate sodium intake (2.9 g/d) in the LN group. Despite no significant differences (*p* = 0.10) between groups, high sodium intake, possibly through the consumption of restaurant or processed foods, may have masked any BP-lowering effects in our HN group [39]. Urinary potassium excretion levels in both groups (~2.8 g/d), however, were consistent with levels observed with the DASH dietary pattern [40], which supports adherence to increased vegetable powder intake in both groups.

Our 4-month study represents one of the longest RCTs to date to investigate the vascular effects of dietary nitrate in individuals with hypertension, following the 6-month trial by Faconti et al. [31,41]. In repeated-measures analysis of BP at two timepoints over 16 weeks, we did not observe differences in BP changes between treatments (time–treatment interaction *p* > 0.05), although differences in BP earlier in the trial are possible. In accordance with several other trials greater than one month in duration [9,31], our study does not support the benefit of chronic dietary nitrate supplementation to BP. Notably, in a preclinical study conducted in rats, chronic sodium nitrate supplementation at a high pharmacological dose was shown to paradoxically elevate BP—a finding accompanied by decreased eNOS activity in the aorta and reduced plasma cGMP, suggesting a net decrease in NO reaching guanylyl cyclase in vascular smooth muscle cells [42]. Given the tight regulation of the endogenous L-arginine-NO production pathway, long-term alternative sources of NO may negatively inhibit eNOS activity, leading to a net decrease in NO formation. Interestingly, in our previous systematic review and meta-analysis of over 40 trials longer than 3 days in duration, we observed that lower doses (<445 mg) were associated with greater treatment effects on systolic BP [43]. While this present clinical trial administered a low dose (400 mg), we did not find any significant treatment differences at 8 and 16 weeks. Future long-term assessments should aim to incorporate more frequent BP measurements at the clinic and at home, using ABPM and home assessments to monitor any decline in efficacy.

Several limitations of the present study should be considered. The trial was statistically underpowered due to disruptions caused by the COVID-19 pandemic. We were unable to reach the originally calculated required sample size of n = 74 (or target enrollment of n = 90 with 15–20% attrition), based on an expected mean difference in office SBP of 7.7 ± 11.74 mmHg between groups [10], as well as an alpha = 0.05 and a 1-Beta = 0.80. Despite a higher attrition rate than anticipated, missing data was similar between groups and 50% of reasons for missing data was due to hospital closures. Therefore, a missing-at-random assumption was made, and multiple imputation was used to fill in missing data in the ITT analysis assessing treatment effects at 16 weeks [44]. Similar findings were observed in the repeated-measures mixed-model approach, which was previously shown to be a statistically powerful model for handling data with missing values in longitudinal studies [20]. Lastly, analyses excluding participants with missing data similarly showed no significant treatment effects.

The absence of an inactive control represents another limitation of this study. A habitual control diet would allow for comparisons in both HN and LN vegetable interventions, providing further insights into the effect size of such dietary interventions. However, a recently published study by Blekkenhorst et al. [25] with such a design did not observe any differences between the consumption of HN vegetables, LN vegetables or no vegetables in individuals with mildly elevated BP. Notably, the nitrate dose was lower (150 mg/day) in that study, and baseline plasma nitrite concentrations (~2.2 μM) were substantially higher than in other studies (<0.5 μM) that found dietary nitrate to be effective [10], indicating the importance of background diets and their contribution to variability between studies. In our study, we instructed participants to avoid mouthwash and non-plant nitrate-/nitrite-rich foods, which represents a potential modification to their habitual diet. While we believed this was an important measure to prevent any interfering effects of mouthwash on nitrate’s conversion to nitrite and contamination with other dietary sources of nitrate/nitrite, our results should be interpreted in the context of these slight modifications to an otherwise habitual diet.

## 5. Conclusions

For individuals with elevated BP, adding HN vegetables (~400 mg) to a regular diet for a sustained period of 16 weeks does not appear to present any greater benefit to BP or arterial stiffness than adding LN vegetables (~50 mg). Given the inconclusive findings to support the benefit of long-term dietary nitrate intake to those with hypertension and the large variability observed in BP response, a confirmatory study is needed that considers dose and duration as important modifiers of treatment efficacy.

## Figures and Tables

**Table 1:** Nutritional composition of high-nitrate and low-nitrate treatments.

Study Intervention	High Nitrate	Low Nitrate
Weight (g)	30.0	30.0
Water (g)	1.9	2.1
Calories (kcal)	86.5	95.1
Carbohydrate (g)	18.7	19.2
Fiber (g)	6.4	5.6
Fat (g)	0.8	1
Protein (g)	5.4	4.7
Nitrate (mg)	404	48
Potassium (mg)	810	660
Sodium (mg)	228	57
Magnesium (mg)	108	42
Calcium (mg)	216	105
Vitamin C (mg)	75.7	104.7

**Table 2:** Baseline characteristics of participants in high-nitrate and low-nitrate treatment groups.

Characteristics	High-Nitrate (n = 33)	Low-Nitrate (n = 33)	*p* Value
Age, years	49.9 (11.8)	53.1 (0.7)	0.23
Male, No. (%)	20 (60.6)	19 (57.6)	
Female, No. (%)	13 (39.4)	14 (42.4)	0.80
Body weight, kg	79.5 (14.2)	81.9 (13.4)	0.48
BMI, kg/m^2^	27.7 (3.3)	28.1 (3.2)	0.59
Body fat, %	29.1 (7.5)	30.2 (8.1)	0.56
Lipids Total Cholesterol, mmol/L	4.9 (1.0)	5.3 (1.1)	0.18
HDL Cholesterol, mmol/L	1.3 (0.3)	1.3 (0.3)	0.59
LDL Cholesterol	3.0 (1.0)	3.3 (1.0)	0.17
Non-HDL Cholesterol, mmol/L	3.6 (1.0)	4.0 (1.1)	0.24
Triglycerides, mmol/L	1.4 (0.7)	1.4 (0.6)	0.91
High-sensitivity C-reactive protein, mg/L	1.5 (1.1)	2.5 (3.0)	0.08
AST, U/L	20.5 (6.4)	21.0 (7.1)	0.77
Creatinine, µmol/L	80.4 (12.1)	71.8 (15.5)	0.02 *
Blood Pressure Office Systolic Diastolic	139.6 (10.0) 88.7 (8.7)	137.7 (11.0) 85.1 (8.6)	0.46 0.10
24-hour ABPM ^a^			
Systolic	130.4 (11.4)	130.6 (9.5)	0.95
Diastolic	81.1 (8.2)	82.4 (7.6)	0.53
Wake ABPM ^b^			
Systolic	134.5 (11.6)	135.2 (12.6)	0.82
Diastolic	84.9 (9.1)	85.5 (8.9)	0.82
Sleep ABPM ^c^			
Systolic	120.6 (12.7)	119.2 (11.8)	0.66
Diastolic	72.5 (8.3)	72.8 (8.5)	0.88
Central			
Systolic	129.0 (9.8)	128.8 (12.8)	0.95
Diastolic	90.0 (8.7)	86.6 (9.1)	0.13
Heart Rate, bpm	66.6 (9.2)	67.9 (9.5)	0.58
Arterial Stiffness			
AIx75, %	22.3 (11.2)	25.1 (10.1)	
Cf-PWV, m/s ^d^	7.22 (3.14)	7.70 (2.86)	0.55
Duration of Elevated BP, months ^e^	39.5 (82.4)	25.8 (88.2)	0.52
Concurrent Medications, No. (%)			
Antihyperglycemic agents	4 (12.1)	3 (9.1)	0.69
Cholesterol-lowering agents	4 (12.1)	4 (12.1)	1.00
Hypothyroid medication	2 (6.1)	1 (3.0)	0.55
Antidepressants	3 (9.1)	1 (3.0)	0.30
Proton pump inhibitor	2 (6.1)	0 (0.0)	0.15
Steroid/bronchodilator	1 (3.0)	1 (3.0)	1.00

Values presented as mean ± SD unless stated otherwise. ^a^ n = 30 in HN group, n = 28 in LN group. ^b^ n = 31 in HN, n = 30 in LN, ^c^ n = 30 in HN, n = 29 in LN, ^d^ n = 27 in HN, n = 28 in LN. ^e^ Duration of high BP is the number of months prior to the screening visit since the participant was first aware or was diagnosed with high blood pressure, defined as >130 mmHg office systolic BP. * *p* < 0.05 as determined by chi-squared test for categorical variables and Student’s *t*-test for continuous variables. ABPM—ambulatory blood pressure monitoring; AST—aspartate aminotransferase; AIx75—augmentation index adjusted to heart rate of 75 beats per minute; BMI—body mass index; bpm—beats per minute; Cf-PWV—carotid–femoral pulse wave velocity; HDL—high-density lipoprotein; LDL—low-density lipoprotein; m/s—meter per second.

**Table 3:** Effects of high-nitrate and low-nitrate vegetable treatments in intention-to-treat analysis.

Outcome		High-Nitrate Within Group				Low-Nitrate Within Group				Between Groups	
		Week 0	Week 16	Δ	*p*	Week 0	Week 16	Δ	*p*	Difference	*p*
Blood Pressure (mmHg)											
Office											
Systolic	66	139.6 ± 1.74	138.53 ± 2.85	−1.30 ± 2.92	0.66	137.7 ± 1.92	134.62 ± 2.65	−4.39 ± 2.19	0.05	3.91 ± 3.52	0.27
Diastolic	66	88.68 ± 1.52	88.39 ± 1.65	−0.94 ± 1.69	0.58	85.13 ± 1.50	86.18 ± 2.04	−1.33 ± 1.53	0.39	2.21 ± 2.09	0.30
24-Hour Ambulatory											
Systolic	66	129.61 ± 2.12	135.61 ± 2.47	1.84 ± 1.86	0.33	131.51 ± 2.26	130.62 ± 3.89	−2.98 ± 2.92	0.33	4.98 ± 3.49	0.18
Diastolic	66	81.00 ± 1.49	84.30 ± 2.36	0.31 ± 1.51	0.84	82.97 ± 1.53	82.91 ± 1.95	−1.07 ± 1.84	0.56	1.39 ± 2.28	0.54
Wake Ambulatory											
Systolic	66	134.52 ± 2.08	139.98 ± 2.55	1.18 ± 2.13	0.58	135.23 ± 2.28	137.24 ± 2.28	−1.08 ± 2.21	0.63	2.74 ± 3.09	0.39
Diastolic	66	84.71 ± 1.80	86.56 ± 1.81	−0.75 ± 1.64	0.65	85.78 ± 1.70	88.10 ± 1.82	0.78 ± 1.93	0.69	−1.53 ± 2.14	0.47
Sleep Ambulatory											
Systolic	66	120.55 ± 2.41	125.80 ± 3.35	3.80 ± 2.71	0.17	120.13 ± 2.49	119.98 ± 3.79	−2.50 ± 3.14	0.43	5.82 ± 3.92	0.14
Diastolic	66	72.42 ± 1.52	74.51 ± 3.84	1.25 ± 2.48	0.62	73.12 ± 1.91	73.33 ± 3.30	−0.36 ± 3.26	0.91	1.18 ± 3.34	0.73
Central											
Systolic	66	128.97 ± 1.71	126.90 ± 2.92	−3.84 ± 2.64	0.15	128.80 ± 2.23	125.67 ± 2.78	−4.17 ± 2.33	0.08	1.23 ± 3.56	0.73
Diastolic	66	89.97 ± 1.52	87.92 ± 2.71	−2.76 ± 2.34	0.25	86.64 ± 1.58	86.59 ± 1.58	−2.48 ± 1.31	0.06	1.34 ± 2.37	0.58
Arterial Stiffness											
Cf-PWV, m/s	66	7.17 ± 0.60	7.90 ± 0.81	0.05 ± 0.75	0.95	7.17 ± 0.60	7.96 ± 0.72	−0.04 ± 0.79	0.96	−0.061 ± 0.73	0.93
AIx75, %	66	22.28 ± 1.95	24.64 ± 1.75	−0.16 ± 1.62	0.92	25.05 ± 1.76	24.90 ± 1.59	−0.38 ± 1.22	0.76	−0.26 ± 1.62	0.87
Lipids (mmol/L)											
Total Cholesterol	66	4.83 ± 0.19	5.24 ± 0.20	0.27 ± 0.18	0.15	5.29 ± 0.20	4.91 ± 0.16	−0.10 ± 0.15	0.48	0.34 ± 0.23	0.16
LDL-Cholesterol	66	2.91 ± 0.17	3.17 ± 0.16	0.16 ± 0.14	0.30	3.27 ± 0.20	3.04 ± 0.13	−0.0017 ± 0.12	0.99	0.13 ± 0.18	0.48
HDL-Cholesterol	66	1.27 ± 0.05	1.23 ± 0.05	−0.03 ± 0.03	0.26	1.30 ± 0.05	1.26 ± 0.04	−0.01 ± 0.03	0.63	−0.03 ± 0.04	0.42
Non-HDL Cholesterol	66	3.60 ± 0.18	3.92 ± 0.21	0.21 ± 0.18	0.26	3.95 ± 0.21	3.63 ± 0.20	−0.10 ± 0.15	0.51	0.29 ± 0.19	0.14
Triglycerides	66	1.42 ± 0.13	1.73 ± 0.21	0.28 ± 0.21	0.19	1.42 ± 0.11	1.36 ± 0.18	−0.04 ± 0.14	0.76	0.38 ± 0.22	0.10
Inflammation											
hs-CRP, mg/L	63	1.58 ± 0.24	1.68 ± 0.30	0.32 ± 0.28	0.27	1.71 ± 0.27	1.16 ± 0.26	−0.29 ± 0.22	0.20	0.52 ± 0.40	0.21

ITT analysis includes all individuals randomized at baseline (n = 66). Multiple imputation was used to fill in missing values. Five imputed datasets were generated. ANCOVA was performed within each dataset and results were pooled together using Rubin’s rule. Individuals with hs-CRP values greater than 10 mg/L were excluded for hs-CRP analysis. Values at 16 weeks and difference estimates between groups represent least-square means and difference in least-square means, respectively, adjusted for center, age, sex, BMI and baseline values. Baseline and within-group change values (Δ) are from paired *t*-test analyses and represent unadjusted values. AIx75—augmentation index adjusted to heart rate of 75 beats per minute; Cf-PWW—carotid–femoral pulse wave velocity; hs-CRP—high-sensitivity C-reactive protein; LDL—low-density lipoprotein; HDL—high-density lipoprotein. * *p*-value < 0.05. Data presented as means ± SE.

**Table 4:** Measures of adherence to study protocol in high-nitrate and low-nitrate groups.

Measures of Adherence	n	High Nitrate	Low Nitrate	*p*
Powder consumed (%)	48	87.5 ± 3.7	92.5 ± 3.6	0.33
Body weight (kg)	48	75.1 ± 2.5	80.6 ± 2.6	0.14
Started antihypertensive medication, n (%)	66	4 (12.1)	1 (3.0)	0.16
24 h urinary sodium excretion				
mmol/d	39	171 ± 22	128 ± 15	0.10
mg/d	39	3937 ± 505	2934 ± 340	0.10
24 h urinary potassium				
mmol/d	36	73 ± 7	72 ± 10	0.99
mg/d	36	2834 ± 277	2830 ± 386	0.99

Values presented were assessed at 16 weeks. *p*-values for test of differences in continuous variables and categorical variables were assessed by Student’s *t*-test and chi-squared test, respectively. Data presented as means ± SE.

## Data Availability

The data supporting the conclusions of this article will be made available by the authors on request.

## Supplementary Materials

The following supporting information can be downloaded at: https://www.mdpi.com/article/10.3390/nu16173018/s1, Table S1: Nutrient intake estimated from three-day food records; Figure S1: Subgroup analysis for the effect of high-nitrate vs. low-nitrate supplementation on office DBP; Figure S2: Subgroup analysis for the effect of high-nitrate vs. low-nitrate supplementation on 24-h ambulatory SBP; Figure S3: Subgroup analysis for the effect of high-nitrate vs. low-nitrate supplementation on 24-h ambulatory DBP; Figure S4: Subgroup analysis for the effect of high-nitrate vs. low-nitrate supplementation on central SBP; Figure S5: Subgroup analysis for the effect of high-nitrate vs. low-nitrate supplementation on central DBP; Figure S6: Subgroup analysis for the effect of high-nitrate vs. low-nitrate supplementation on AIx75; Figure S7: Subgroup analysis for the effect of high-nitrate vs. low-nitrate supplementation on cf-PWV.
