# Supplementary material for: No Difference between the Efficacy of High-Nitrate and Low-Nitrate Vegetable Supplementation on Blood Pressure after 16 Weeks in Individuals with Early-Stage Hypertension: An Exploratory, Double-Blinded, Randomized, Controlled Trial
Source: Nutrients. 2024 Sep 6;16(17):3018. doi: 10.3390/nu16173018 (PMC11397180; doi:10.3390/nu16173018)
Supplement: Supplementary file 1 [file nutrients-16-03018-s001.zip › nutrients-3180199-supplementary.pdf]

Supplementary Material

Table S1. Nutrient intake estimated from 3-day food records.

Figure S1. Subgroup analysis for the effect of high-nitrate vs. low-nitrate supplementation on office DBP

Figure S2. Subgroup analysis for the effect of high-nitrate vs. low-nitrate supplementation on 24-hour ambulatory SBP

Figure S3. Subgroup analysis for the effect of high-nitrate vs. low-nitrate supplementation on 24-hour ambulatory DBP

Figure S4. Subgroup analysis for the effect of high-nitrate vs. low-nitrate supplementation on central SBP

Figure S5. Subgroup analysis for the effect of high-nitrate vs. low-nitrate supplementation on central DBP

Figure S6. Subgroup analysis for the effect of high-nitrate vs. low-nitrate supplementation on AIx75

Figure S7. Subgroup analysis for the effect of high-nitrate vs. low-nitrate supplementation on cf-PWV

Table S1. Nutrient intake estimated from 3-day food records.

Daily Intake	High-Nitrate Group	Low-Nitrate Group	*P*
Energy (kcal)	1846 ± 1008	1729 ± 646	0.65
Carbohydrates (% of total kcal)	48.4 ± 14.6	47.2 ± 12.7	0.78
Protein (% of total kcal)	19.4 ± 5.9	19.7 ± 8.0	0.91
Fat (% of total kcal)	32.6 ± 10.3	31.8 ± 10.6	0.81
Saturated Fat (% of total kcal)	10.3 ± 4.5	9.1 ± 3.9	0.37
Polyunsaturated Fat (% of total kcal)	4.2 ± 2.6	4.0 ± 2.5	0.80
Monounsaturated Fat (% of total kcal)	7.5 ± 5.2	6.3 ± 4.4	0.43
Cholesterol (mg)	323 ± 260	227 ± 162	0.17
Fibre (g)	17.3 ± 8.0	22.9 ± 14.3	0.11
Potassium (mg)	1543 ± 743	1342 ± 718	0.37
Sodium (mg)	2609 ± 1636	2333 ± 1315	0.54
Magnesium (mg)	174 ± 115	181 ± 24	0.85
Calcium (mg)	642 ± 471	560 ± 254	0.49
Vitamin C (mg)	87 ± 102	74 ± 14	0.62

Nutrient analyses were calculated from the average of 3-day food records collected at the final study visit (Week 16). Presented data are from participants who completed the study and completed a food record at the final study visit (n=43). Data represented as means ± SD.

Subgroups	N	Mean Difference [95% CIs] (mmHg)	*p* value
**Overall**	48	2.04 [-2.14,6.22]	0.33
**Centre**			
**Toronto**	40	1.55 [-3.25,6.35]	0.75
**Zagreb**	8	3.46 [-8.94,15.86]	
**Age (years)**			
**<60**	32	4.59 [-0.68,9.85]	0.09
**≥60**	16	-3.61 [-11.9,4.68]	
**Sex**			
**Female**	24	3.68 [-2.53,9.88]	0.36
**Male**	24	-0.23 [-6.31,5.85]	
**BMI (kg/m²)**			
**<25**	7	-4.34 [-17.07,8.39]	0.43
**25-29.9**	8	3.46 [-2.51,9.43]	
**≥30**	33	3.14 [-9.08,15.36]	
**Baseline DBP (mmHg)**			0.94
**<90**	30	2.15 [-3.21,7.51]	
**≥90**	18	2.49 [-4.66,9.64]	

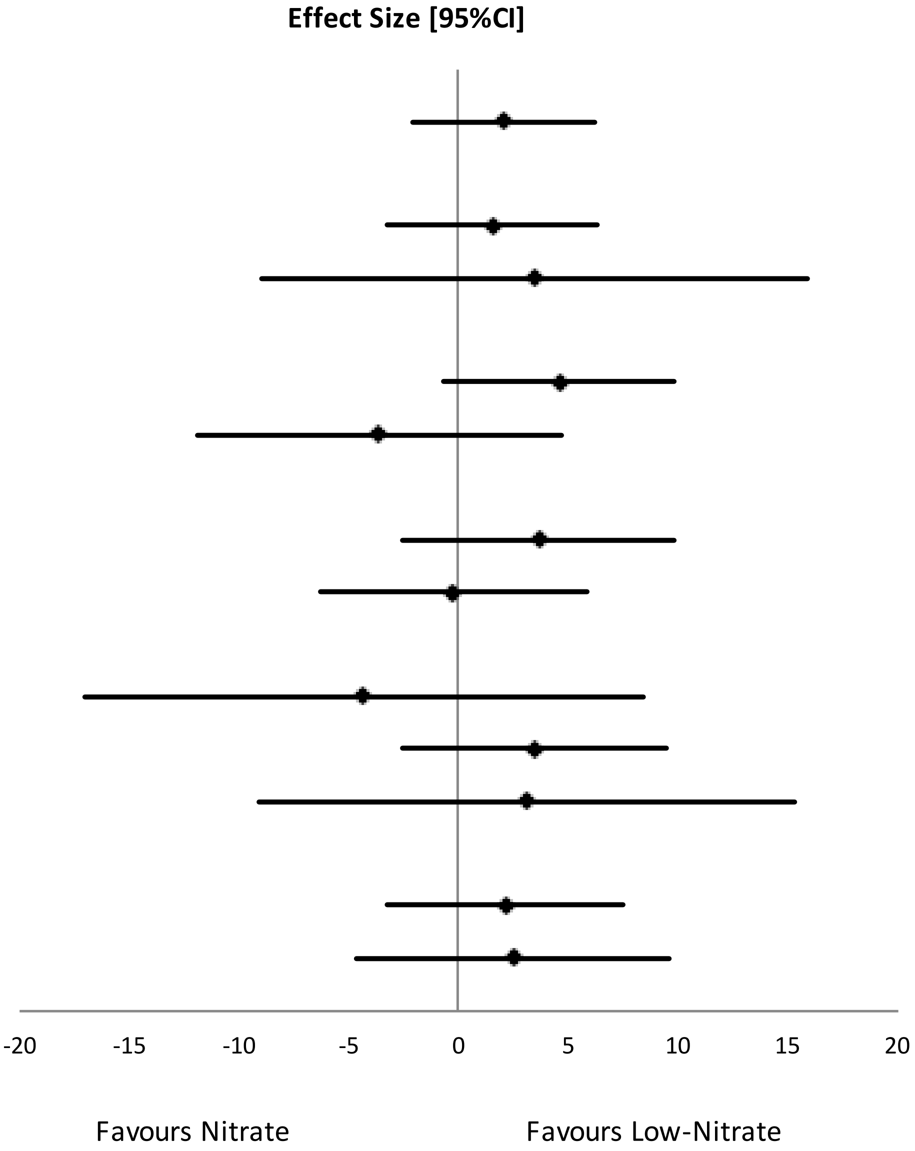

Figure S1: Subgroup analysis for the effect of high-nitrate vs. low-nitrate supplementation on office DBP, in completers (n=48). Mean differences with 95% CIs represent the treatment difference between groups within each subgroup category, adjusted for centre and baseline value, unless not applicable. *P*-values represent test for treatment*subgroup interaction from ANCOVA. BMI- body mass index; CI-confidence interval; SBP- systolic blood pressure.

Subgroups	N	Mean Difference [95% CIs] (mmHg)	Effect Size [95%CI]	*p* value
**Overall**	40	4.33 [-1.03,9.69]		0.11
**Centre**				
**Toronto**	34	5.89 [-0.3,12.09]		0.25
**Zagreb**	6	-3.79 [-22.97,15.4]		
**Age (years)**				
**<60**	28	4.13 [-3.24,11.5]		0.71
**≥60**	12	6.56 [-5.58,18.7]		
**Sex**				
**Female**	20	2.81 [-4.77,10.4]		0.65
**Male**	20	5.22 [-2.75,13.18]		
**BMI (kg/m²)**				
**<25**	6	-10.01 [-27.35,7.32]		0.10
**25-29.9**	7	5.71 [-2.81,14.23]		
**≥30**	27	10.36 [-5.07,25.79]		
**Baseline 24hr SBP (mmHg)**				0.74
**<130**	23	1.66 [-8.8,12.13]		
**≥130**	17	4.26 [-8.54,17.06]		

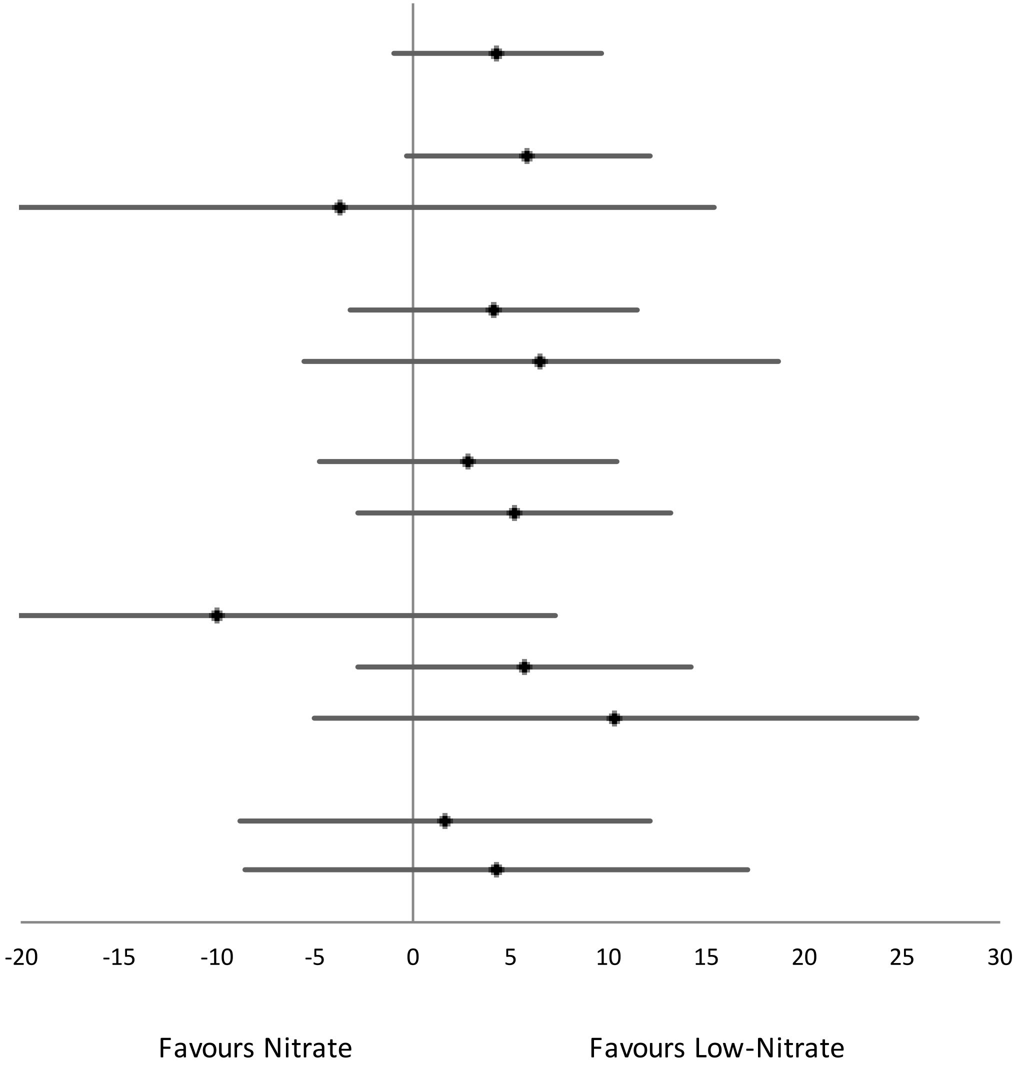

Figure S2: Subgroup analysis for the effect of high-nitrate vs. low-nitrate supplementation on 24-hour SBP, in completers (n=40). Mean differences with 95% CIs represent the treatment difference between groups within each subgroup category, adjusted for centre and baseline value, unless not applicable. *P*-values represent test for treatment*subgroup interaction from ANCOVA. BMI- body mass index; CI-confidence interval; SBP- systolic blood pressure.

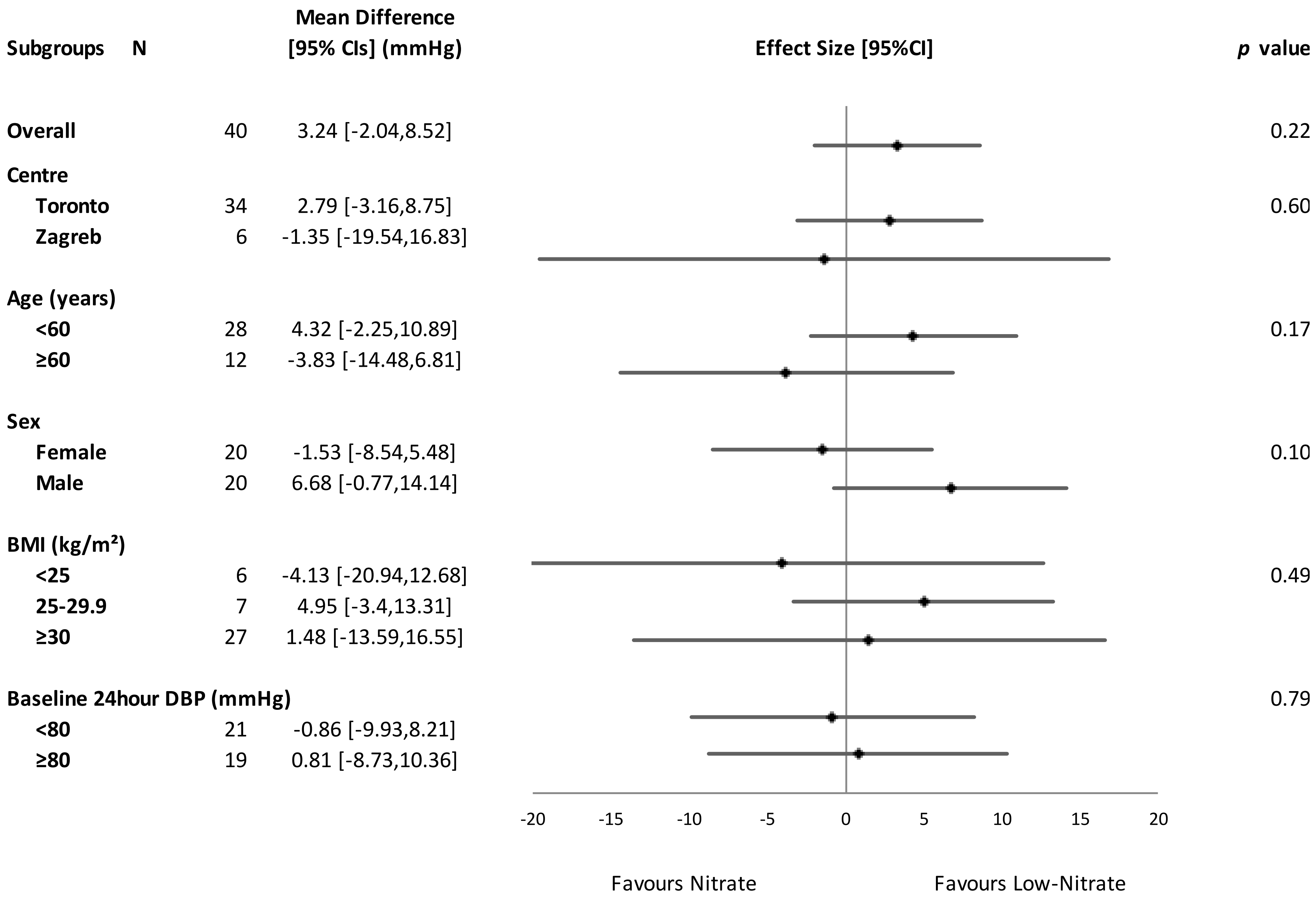

Figure S3. Subgroup analysis for the effect of high-nitrate vs. low-nitrate supplementation on 24-hour DBP, in completers (n=40). Mean differences with 95% CIs represent the treatment difference between groups within each subgroup category, adjusted for centre and baseline value, unless not applicable. *P*-values represent test for treatment*subgroup interaction from ANCOVA. BMI- body mass index; CI-confidence interval; SBP- systolic blood pressure.

Subgroups	N	Mean Difference [95% CIs] (mmHg)	*p* value
**Overall**	46	2.69 [-4.4,9.78]	0.45
**Centre**			
**Toronto**	38	2.72 [-5.2,10.65]	0.76
**Zagreb**	8	-0.22 [-20.73,20.28]	
**Age (years)**			
**<60**	31	4.21 [-4.51,12.92]	0.15
**≥60**	15	-7.04 [-20.49,6.41]	
**Sex**			
**Female**	23	4.4 [-5.84,14.65]	0.47
**Male**	23	-0.75 [-11.12,9.61]	
**BMI (kg/m²)**			
**<25**	6	-9.52 [-33.27,14.24]	0.50
**25-29.9**	7	1.44 [-8.02,10.9]	
**≥30**	33	5.51 [-13.83,24.85]	
**Baseline Central SBP (mmHg)**			0.25
**<130**	25	-2.89 [-14.75,8.96]	
**≥130**	21	7.22 [-6.02,20.47]	

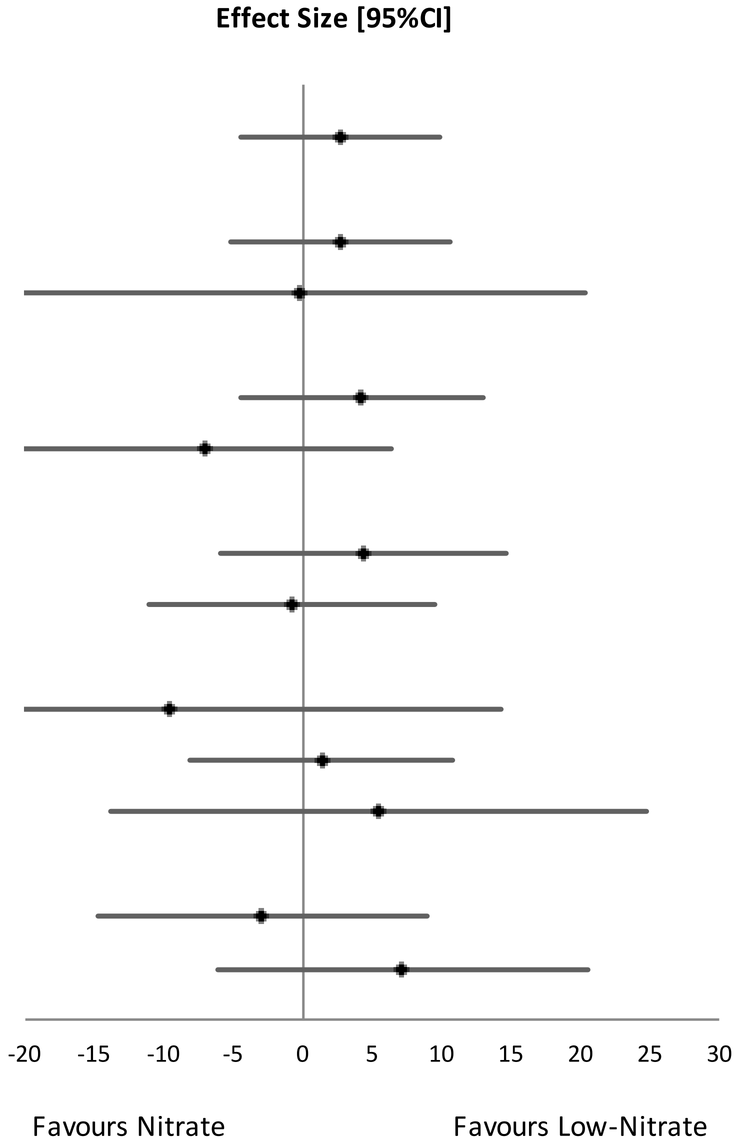

Figure S4: Subgroup analysis for the effect of high-nitrate vs. low-nitrate supplementation on central SBP, in completers (n=46). Mean differences with 95% CIs represent the treatment difference between groups within each subgroup category, adjusted for centre and baseline value, unless not applicable. *P*-values represent test for treatment*subgroup interaction from ANCOVA. BMI- body mass index; CI-confidence interval; SBP- systolic blood pressure.

Subgroups	N	Mean Difference [95% CIs] (mmHg)	Effect Size [95%CI]	*p* value
**Overall**	46	2.14 [-1.95,6.23]		0.30
**Centre**				
**Toronto**	38	2.31 [-2.45,7.08]		0.94
**Zagreb**	8	1.9 [-10.1,13.9]		
**Age (years)**				
**<60**	31	5.38 [0.23,10.53]		0.06
**≥60**	15	-3.49 [-11.93,4.94]		
**Sex**				
**Female**	23	3.97 [-2.2,10.15]		0.35
**Male**	23	0.04 [-6.1,6.18]		
**BMI (kg/m²)**				
**<25**	6	-4.83 [-19.27,9.62]		0.43
**25-29.9**	7	2.48 [-3.45,8.41]		
**≥30**	33	5.39 [-6.73,17.5]		
**Baseline Central DBP (mmHg)**				0.77
**<90**	28	3.03 [-2.37,8.44]		
**≥90**	18	1.8 [-5.14,8.75]		

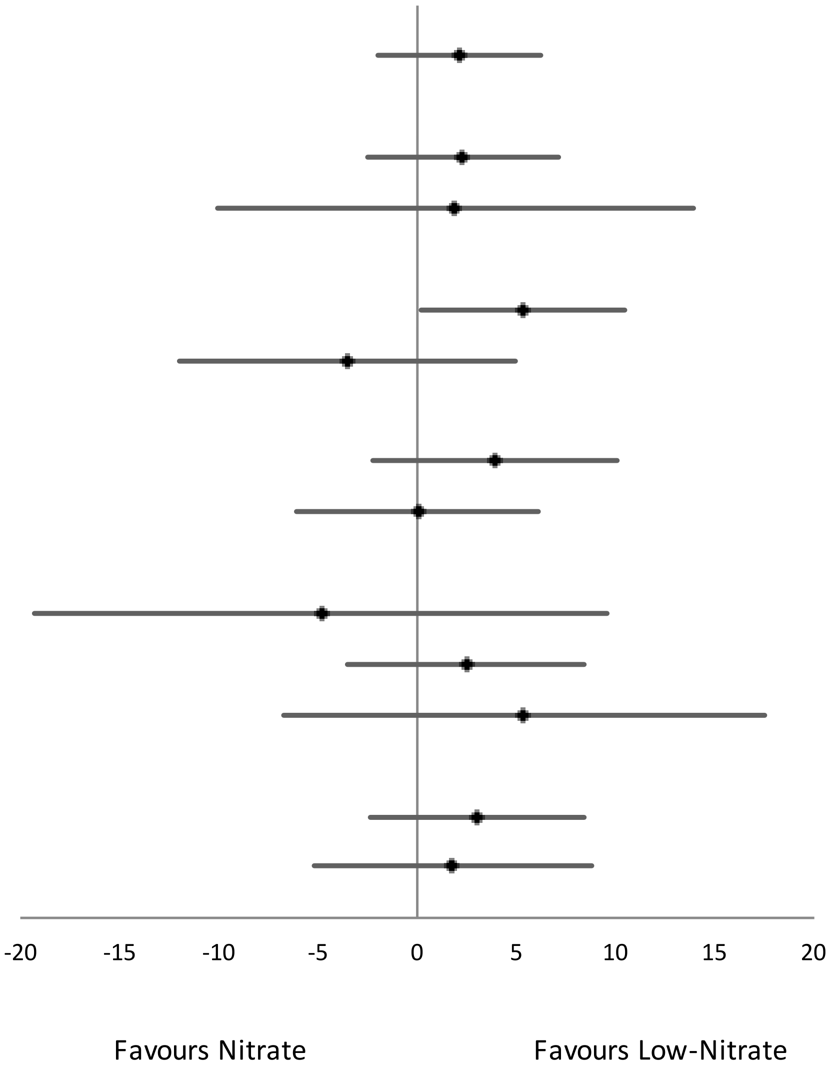

Figure S5: Subgroup analysis for the effect of high-nitrate vs. low-nitrate supplementation on central DBP, in completers (n=46). Mean differences with 95% CIs represent the treatment difference between groups within each subgroup category, adjusted for centre and baseline value, unless not applicable. *P*-values represent test for treatment*subgroup interaction from ANCOVA. BMI- body mass index; CI-confidence interval; SBP- systolic blood pressure.

Subgroups	N	Mean Difference [95% CIs] (%)	*p* value
**Overall**	46	1.08 [-2.46,4.62]	0.54
**Centre**			
**Toronto**	38	1.78 [-2.5,6.06]	0.86
**Zagreb**	8	0.86 [-10.08,11.8]	
**Age (years)**			
**<60**	31	2.07 [-2.53,6.66]	0.18
**≥60**	15	-3.3 [-10.31,3.71]	
**Sex**			
**Female**	23	-1.26 [-6.54,4.02]	0.24
**Male**	23	3.03 [-2.28,8.34]	
**BMI (kg/m²)**			
**<25**	6	4.96 [-9.37,19.29]	0.76
**25-29.9**	7	0.8 [-4.91,6.51]	
**≥30**	33	3.93 [-8.03,15.9]	
**Baseline AIx75 (%)**			0.23
**<25.4**	19	5.71 [-1.15,12.56]	
**≥25.4**	27	0.45 [-5.23,6.14]	

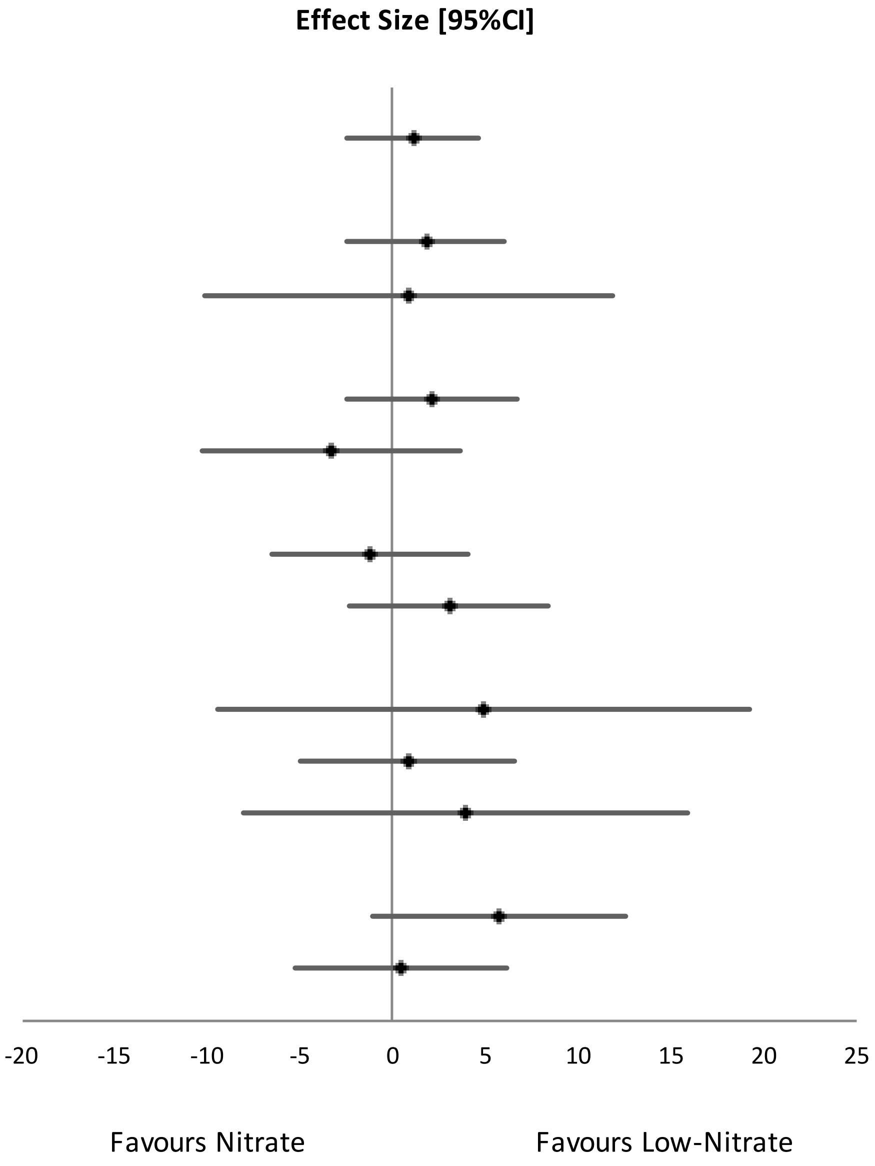

Figure S6: Subgroup analysis for the effect of high-nitrate vs. low-nitrate supplementation on central AIx75, in completers (n=46). Mean differences with 95% CIs represent the treatment difference between groups within each subgroup category, adjusted for centre and baseline value, unless not applicable. *P*-values represent test for treatment*subgroup interaction from ANCOVA. BMI- body mass index; CI-confidence interval; SBP- systolic blood pressure.

Subgroups	N	Mean Difference [95% CIs] (m/s)	*p* value
**Overall**	37	0 [-1.95,1.94]	1.00
**Centre**			
**Toronto**	29	-0.12 [-2.26,2.03]	0.90
**Zagreb**	8	-0.41 [-5.14,4.33]	
**Age (years)**			
**<60**	25	-0.94 [-3.22,1.35]	0.25
**≥60**	12	1.33 [-2.1,4.76]	
**Sex**			
**Female**	23	0.76 [-1.63,3.15]	0.20
**Male**	14	-1.71 [-4.9,1.49]	
**BMI (kg/m²)**			
**<25**	5	-3.04 [-9.48,3.39]	0.54
**25-29.9**	6	-0.02 [-2.7,2.66]	
**≥30**	26	-0.03 [-5.06,5]	
**Baseline cf-PWV (m/s)**			0.21
**<7.4**	28	-0.66 [-2.97,1.64]	
**≥7.4**	9	2.63 [-2.49,7.75]	

Effect Size [95%CI]

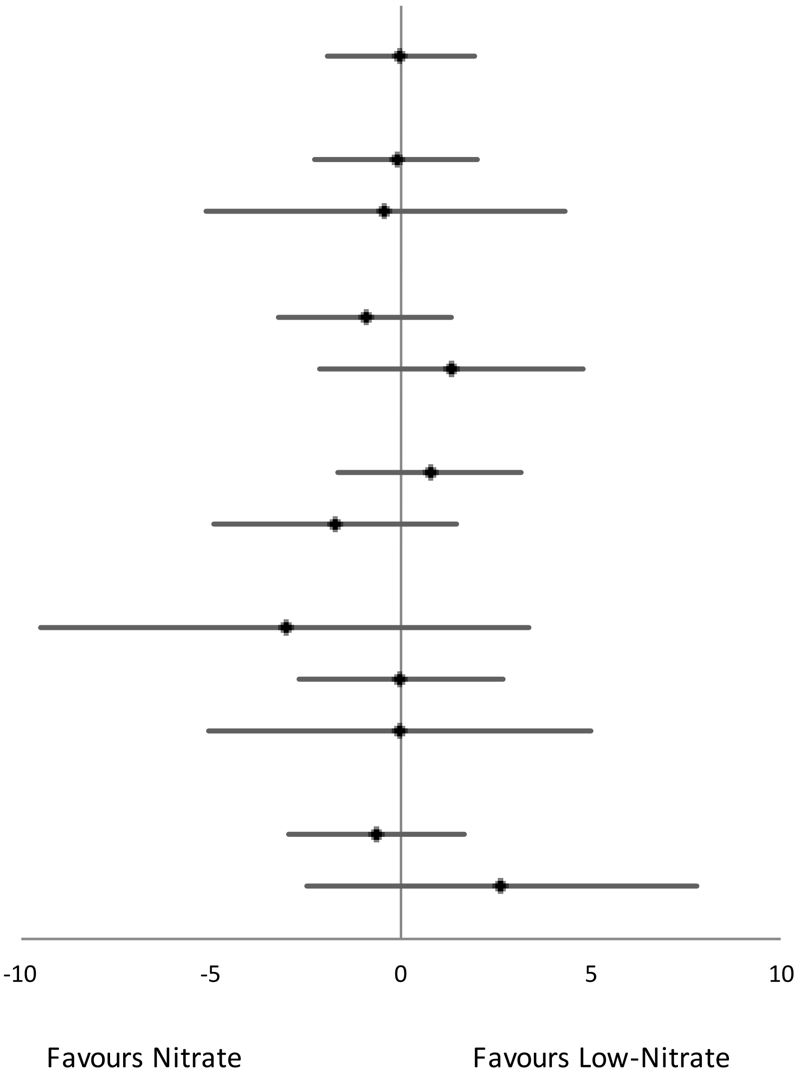

Figure S7: Subgroup analysis for the effect of high-nitrate vs. low-nitrate supplementation on PWV, in completers (n=37). Mean differences with 95% CIs represent the treatment difference between groups within each subgroup category, adjusted for centre and baseline value, unless not applicable. *P*-values represent test for treatment*subgroup interaction from ANCOVA. BMI- body mass index; CI-confidence interval; SBP- systolic blood pressure
